# MORPHOLOGICAL ANALYSIS OF THE SCAPULA AND ITS IMPLICATIONS IN BRISTOW-LATARJET PROCEDURE

**DOI:** 10.1590/1413-785220172501161719

**Published:** 2017

**Authors:** JOANA DANIELA DE OLIVEIRA SILVA, CATARINA NEVES DAMAS, MÁRCIA CHRISTEL DE CARVALHO SÁ, JOÃO MANUEL COSTA FERREIRA TORRES

**Affiliations:** 1. University of Porto, Faculty of Medicine, Hospital S. João, Porto, Portugal; 2. Primary Healthcare Unit "Saúde em Família". Pedrouços, Maia, Portugal.

**Keywords:** Shoulder dislocation/etiology. Joint instability. Range of motion, articular. Treatment outcome

## Abstract

**Objective:**

To assess which of two procedures, Bristow or Latarjet, is anatomically the most appropriate for the general population***.***

**Methods:**

One thousand one hundred and thirty two shoulders were evaluated by an observer who measured the following coracoid process parameters - length, angle and minimum thickness - through Computed Tomography (CT) analysis. Statistical analysis was carried out by ANOVA and Bland-Altman tests***.***

**Results:**

The mean length, angle and minimum thickness of the coracoid were 27.0 ± 3.80 mm; 103.54 ± 14.03°; and 9.16 ± 6.38 mm, respectively. Gender differences were statistically significant***.***

**Conclusion:**

According to this image-based anatomic study, the coracoid process dimensions do not influence the choice between Bristow or Latarjet procedures. ***Level of Evidence III, Therapeutic Studies - Investigating the Results of Treatment.***

## RESUMO

**Objetivo:**

***Avaliar qual dos dois procedimentos, Bristow ou Latarjet, é o mais adequado anatomicamente para a população em geral.***

**Métodos:**

***Um mil cento e trinta e dois ombros foram sujeitos à avaliação de vários parâmetros do processo coracóide - comprimento, ângulo e espessura mínima - através da análise de exames de Tomografia Computadorizada (TC). A análise estatística teve por base os testes ANOVA e Bland-Altman.***

**Resultados:**

***As médias obtidas do comprimento, do ângulo e da espessura mínima do processo coracóide foram, respetivamente, 27,00 ± 3,80 mm, 103,54 ± 14,03° e 9,16 ± 6,38 mm. As diferenças obtidas entre gêneros foram estatisticamente significativas.***

**Conclusões:**

*De acordo com o estudo realizado, as dimensões do processo coracóide não constituem um critério para a decisão da opção terapêutica entre os procedimentos em causa, Latarjet e Bristow.* ***Nível de Evidência III, Estudos Terapêuticos - Investigação dos Resultados do Tratamento.***

## INTRODUCTION

The shoulder is the most mobile joint of the human body; as a result of its wide range of movement, the glenohumeral joint is highly susceptible to dislocation. This common injury^1^ represents 50% of all joint dislocations.^2^ Young men who sustain high-energy injuries to the shoulder are most affected.^3^ This condition often occurs in athletes and peaks in the second and sixth decades of life.^4^

Previous studies have shown that coracoid transfer procedures are biomechanically advantageous over other glenoid reconstruction options such as autograft from the iliac crest or the use of allografts because of the additional dynamic stabilizing ''sling'' effect produced by the repositioned conjoint tendon. Consequently, coracoid transfer is considered a good solution for instability-related glenoid defects and even isolated capsulolabral tears.^5^

Two common treatments for anterior dislocations are the Latarjet and Bristow procedures. In the Latarjet procedure the entire coracoid process is transferred so that the inferior surface follows the curved shape of the glenoid and is fixed with two screws, while in the Bristow procedure only the tip of the coracoid process is transferred and is fixed with a single screw to the resected surface in contact with the glenoid.^6^ ^-^ ^8^

Since both of these techniques consist of coracoid transfer procedures and a significant proportion of patients with this pathology will require surgery,^3^ we believe it is important to study the anatomy of the coracoid process to determine which of these two procedures is the most anatomically appropriate for the general population. In this study we measured the length, angle, and minimum thickness of this process.

## MATERIALS AND METHODS

Chest CT scans performed at a central hospital for diagnostic purposes during two randomly chosen months (June 9-August 8, 2014) were evaluated by an observer with a master's degree. A total of 566 CT scans were obtained (1132 shoulders). Sixty-six CT scans (11.66%) in which it was not possible to measure the coracoid process were excluded. Exclusion criteria were: repeated subject (N=1, 0.18%), CT scans that did not show the coracoid process (N=54, 9.52%), presence of cartilage growth (N=8; 1.41%), and presence of degenerative changes (N=4; 0.70%). (Table 1) Other exclusion criteria were cases with a history of bone surgery or scapula fracture, but no patients presented these characteristics.

**Table 1:** Exclusion Criteria.

Exclusion Criteria		
Criteria	Frequency (n)	Percent (%)
Presence of degenerative changes	4	0.70
Presence of growth cartilage	8	1.41
Coracoid process not visible in the CT scans	54	9.52
Repeated subject	1	0.18
Total	66	11.81

**Figure 1:**
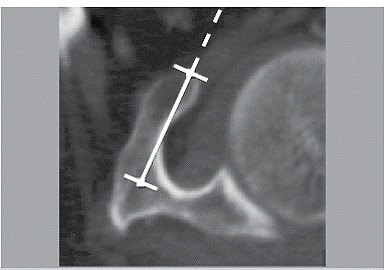
Coracoid length.

**Figure 2:**
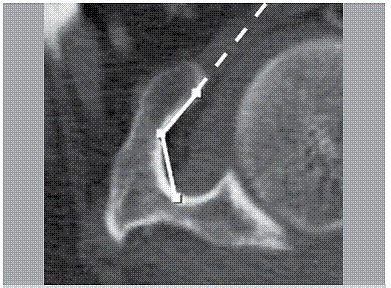
Coracoid angle.

**Figure 3:**
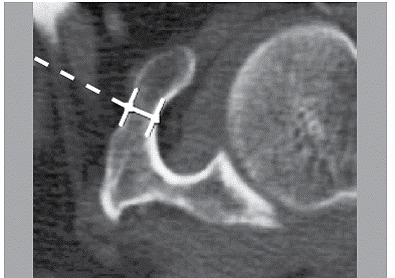
Minimum thickness of the coracoid process.

The CT scans were accessed and targeted parameters were measured using a Sectra IDS7 workstation, version 15.1.24.1 @2012 Sectra AB. A total of 500 (88.34%) CT scans in the axial plane were reviewed. In all of these CT scans the coracoid process parameters were measured (length, angle, and minimum thickness). Length was defined as the distance between one point on the apex and another point at the base of the coracoid process. (Figure 1) Four points along the lateral border of the coracoid process demarcated the angle: one at the front end of the medial border, the second and the fourth at the point with the greater curvature, and the third at the base of the coracoid process. (Figure 2) Finally, minimum thickness was defined as the shortest distance between two opposite points on the medial and lateral cortical margins of the coracoid body. (Figure 3)

Statistical analysis was performed using Microsoft Excel 2010 and MedCalc 14.12.0 version software. ANOVA was used to analyze the variance between measurements obtained by two researchers working independently. No statistically significant difference was seen between the two researchers, giving power to the method used. Blinded measurements of the two observers and Bland-Altman analysis determined inter-rater reliability. Differences were considered statistically significant at P<0.05. 

Institutional review board approval was not necessary since we did not intervene in patient care or handle personal data. 

## RESULTS 

The CTs from the 500 included subjects (1000 shoulders) comprised 196 (39.20%) female and 304 male (60.80%) subjects with a mean age of 64.15 years (21-95) and 63.64 years (16-93), respectively (IC 95%; P=0.615). (Table 2)

**Table 2:** Included subjects.

Included subjects		
Gender	Frequency	Percent (%)
Female	196	39.20
Male	304	60.80
Total	500	88.34

Length: the length was measured in 864 coracoid processes. Mean length was 27.00 ± 3.80 mm, (Table 3) and minimum and maximum values were 17.70 mm and 40.50 mm, respectively. These included 343 female (25.08 ± 2.98 mm) and 521 male (28.25 ± 3.76 mm) subjects (IC 95%; P<0.0001). (Table 4)

**Table 3:** Data Summary - measurements of the coracoid process.

Parameters	Mean	SD	Minimum	Maximum
Length (mm)	27.00	3.80	17.70	40.05
Angle (º)	103.54	14.03	58.10	155.30
Minimum thickness (mm)	9.16	6.38	5.20	15.80

**Table 4:** Sex differences.

Sex differences				
Parameters	Gender	Mean	SD	Minimum	Maximum	P value
Length (mm)	Female	25,08	2,98	17,70	35,7	< 0,0001
	Male	28,25	3,76	18,40	40,50	
Angle ^(o)^	Female	101,33	14,26	58,1	143,40	0,0001
	Male	104,96	13,70	62,8	155,30	
Minimum thickness (mm)	Female	8,38	6,53	5,20	15,80	0,003
Male	9,67	6,24	5,50	14,4

Angle: the angle was measured in 917 coracoid processes. The mean angle was 103.54 ± 14.03º, (Table 3) and the minimum and maximum values were 58.1º and 155.30º, respectively. These included 357 female (101.33 ± 14.26º) and 560 male (104.96 ± 13.70º) subjects (IC 95%; P=0.0001). (Table 4) 

Minimum thickness: minimum thickness was measured in 916 coracoid processes; the mean minimum thickness was 9.16 ± 6.38 mm, (Table 3) and the minimum and maximum values were 5.20 mm and 15.80 mm, respectively. The subjects were 362 females (8.38 ± 6.53 mm) and 554 males (9.67 ± 6.24 mm) (IC 95%; P=0.003)*.* (Table 4)

Gender differences were statistically significant; as expected, scans of the female subjects exhibited lower mean lengths and minimum thickness. (Table 4) 

## DISCUSSION

Since the dimensions of the shoulder blades vary according to different populations, one limitation of this study is that the sample population may not be representative of the global population. Additionally, since the CT scans were not performed to evaluate the shoulder blade, not all scans allowed us to properly evaluate the coracoid. 

Other studies have quantified the size of the coracoid process; none were carried out expressly for this purpose, however, and do not contain as many cases as we evaluated in this study.

In previous studies, the results for the length of the coracoid process varied significantly according to the type of assessment: namely, measurements taken from cadavers varied more than measurements from studies involving x-rays, which presented values closer to those obtained in this study.^5^ ^,^ ^9^ ^,^ ^10^The minimum thickness of the coracoid process did not vary as much and were similar to our findings.^5^ ^,^ ^9^ ^,^ ^10^ No comparable studies measured the angle of the coracoid process using the definition applied in this study.

The most commonly used screws in the Latarjet or Bristow procedures are 35-mm 4.5-mm partially threaded malleolar screws.^11^ Considering these screw dimensions and the mean, maximum, and minimum values for length and minimum thickness found in this study, we can conclude that the coracoid process demonstrated thickness sufficient to support 1 screw and length sufficient to support 2 screws. Characterization of the angle of the coracoid process is important, since it permits a three-dimensional concept during the surgery; consequently, it appears that the Latarjet or Bristow procedures can be formed interchangeably based on these parameters. 

A low rate of recurrence using different variations of the Latarjet or Bristow procedures was reported in several studies^12^ ^-^ ^17^ which also demonstrated favorable long-term results.^7,10,12^ Coracoid transfer procedures are especially indicated in recurrent anterior dislocations associated with hyperlaxity or glenoid bone loss. In situations featuring voluntary anterior instability or anterior instability without a Bankart lesion these procedures are not recommended. Two common complications of these procedures are non-union of the coracoid process (more common in the Bristow procedure due to the instability resulting from single screw fixation and less bone contact between surfaces) and intra-articular positioning of the graft (more common in the Latarjet procedure due to the larger size of the graft).^18^

Considering that this study revealed that the dimensions of the coracoid process are not relevant criteria for selecting the best surgical option, it seems important to study the glenoid anatomy before choosing a procedure; anterior shoulder instability is frequently associated with glenoid bone loss, which varies greatly in both extent and significance.^11^ ^,^ ^19^ ^-^ ^21^

## CONCLUSION

This image-based anatomic study shows that the dimensions of the coracoid process do not determine whether the Latarjet or Bristow procedures are better choices for surgical treatment. The glenoid anatomy is an important target for further study.
